# Supplementary material for: Exploring Food Addiction Across Several Behavioral Addictions: Analysis of Clinical Relevance
Source: Nutrients. 2025 Apr 6;17(7):1279. doi: 10.3390/nu17071279 (PMC11990926; doi:10.3390/nu17071279)
Supplement: Supplementary file 1 [file nutrients-17-01279-s001.zip › Supplementary Material.pdf]

Supplementary Material

Supplementary Table

Table S1 Consistency in the study

SCL Somatization	.914	UPPS-P Lack of premeditation	.874	TCI-R Novelty seeking	.706
SCL Obsess-compulsive	.883	UPPS-P Lack of perseverance	.802	TCI-R Harm avoidance	.740
SCL Interp.sensitivity	.876	UPPS-P Sensation seeking	.863	TCI-R Reward dependence	.620
SCL Depression	.932	UPPS-P Positive urgency	.929	TCI-R Persistence	.897
SCL Anxiety	.918	UPPS-P Negative urgency	.838	TCI-R Self-directedness	.823
SCL Hostility	.842	UPPS-P Total score	.905	TCI-R Cooperativeness	.730
SCL Phobic anxiety	.874	DERS Non acceptance	.915	TCI-R Self-transcendence	.835
SCL Paranoid ideation	.795	DERS Goal directed behaviors	.806		
SCL Psychotic ideation	.852	DERS Diff. in impulse control	.896		
SCL Global indexes	.981	DERS Lack of awareness	.793		
YFAS-2	.907	DERS Limited access emot.	.885		
		DERS Lack of emotional clarity	.831		
		DERS Total score	.935		
